# Endovascular repair of bilateral isolated common iliac artery aneurysms with unsuitable anatomy utilizing an aortic bifurcated unibody endograft and modified sandwich technique to preserve pelvic blood flow: a case series

**DOI:** 10.1186/s13019-024-02674-2

**Published:** 2024-04-15

**Authors:** Haodong Liao, Enquan Zhou, Yongjiang Tang, Chunshui He

**Affiliations:** 1 Department of Vascular Surgery, Hospital of Chengdu University of Traditional Chinese Medicine, Chengdu, 610072 Sichuan, CN China; 2 Department of Radiology, Hospital of Chengdu University of Traditional Chinese Medicine, Chengdu, Sichuan, CN China; 3 Department of Interventional Radiology, Hospital of Chengdu University of Traditional Chinese Medicine, Chengdu, Sichuan, CN China; 4 Department of Vascular Disease, Panzhihua Municipal Central Hospital, Panzhihua, Sichuan, CN China

**Keywords:** Endovascular repair, Isolated common iliac artery aneurysm, Internal iliac artery, Modified sandwich technique, Case report

## Abstract

Bilateral isolated common iliac artery aneurysms (CIAAs) are rare, and endovascular repair of CIAAs has emerged as an alternative to traditional open surgical repair. The primary goal of therapy is to exclude the aneurysm sac while maintaining perfusion of at least one internal iliac artery (IIA) to prevent pelvic ischemia. Although the iliac branch device (IBD) has improved the feasibility of preserving the IIA, its applicability is limited to a specific subset of aneurysm anatomy. We present a case series of three patients with bilateral isolated CIAAs in whom preoperative CT scans revealed an absence of a landing zone, the diameter of proximal CIA diameter was less than 13.0 mm, and normal diameter of the nonaneurysmal infrarenal aorta, making it challenging to use an IBD alone or a standard bifurcated aortic endograft to provide a proximal landing zone for iliac artery stenting. To overcome the small diameter of the infrarenal aorta, we implanted an aortic bifurcated unibody endograft. Then, we utilized a balloon-expandable covered stent-graft with overdilation as a modified sandwich technique to create an “eye of the tiger” configuration to prevent gutter leakage. The final angiography performed during the procedure revealed successful exclusion of the aneurysms, with blood flow to the right IIA and no type III endoleak. During the postoperative follow-up period, no patients exhibited symptoms associated with pelvic ischemia. There were no endoleaks or sac expansions on the two-year follow-up CT scans, and all external and internal iliac graft limbs were patent. This study demonstrated that a combination of an aortic bifurcated unibody endograft and a modified sandwich technique can effectively treat bilateral isolated CIAAs with certain anatomical constraints.

## Introduction

The occurrence of isolated common iliac artery aneurysms (CIAAs) is rare, accounting for only 2–7% of all intra-abdominal aneurysms. Up to 50% of cases may involve bilateral disease [1]. Isolated CIAAs are characterized by a specific criterion: a twofold enlargement of the common iliac artery (CIA) without the presence of an aneurysm in any other location [2]. The presence of a CIAA is correlated with an elevated risk of rupture, especially when the diameter is greater than 3.5 cm [1], which can have potentially lethal consequences. The established criteria for the treatment of isolated CIAAs typically include aneurysms with a diameter exceeding 3.5 cm, a diameter increase of more than 6 mm within a 6-month period, or an annual diameter increase of over 1 cm [1, 3].

The options for treating CIAAs include open surgery, hybrid procedures, and endovascular therapy. Open surgical repair is regarded as the gold standard for younger and more physically capable patients. However, given the late presentation of the pathology and the complexity of the procedure, this is frequently technically challenging. This may account for the higher mortality rate associated with CIAA repairs compared to AAA repairs, which ranges from 5 to 11%, whereas the mortality rate for an emergency operation following a rupture is between 40 and 50%[4–6].

In recent years, endovascular aneurysm repair (EVAR) of isolated CIAAs has evolved, and the primary goal of therapy is to exclude the aneurysm sac while maintaining at least one IIA perfusion to prevent complications such as buttock claudication, sexual dysfunction, gluteal region necrosis, and colon or spinal cord ischemia [1]. Occasionally, preserving only one IIA may heighten the likelihood of buttock claudication in comparison to preserving both IIAs [7, 8]. IBDs have been developed and are being utilized more frequently for this purpose. However, according to one study, only 35% of patients with CIAA have vascular anatomy appropriate for repair with an IBD [9].

Aegis™ (MicroPort Endovastec, Shanghai, China) is a unique aortic bifurcated unibody endograft that fits the small diameter of the infrarenal aorta and provides a proper landing zone for the CIA [10]. LifeStream™ (Bard Peripheral Vascular, Tempe, USA) is a balloon-expandable covered stent-graft that has the potential to be overdilated [11]. Fluency™ (Bard Peripheral vascular, Tempe, US) is a self-expandable covered stent-graft that can be used in a modified sandwich technique with LifeStream™.

In this study, we present a series of cases involving bilateral isolated CIAAs. These cases were characterized by proximal CIA anatomical features that rendered them unsuitable for the use of an IBD, as well as a narrow distal aorta that posed challenges for standard aortic endograft implantation. Thus, we utilized Aegis™ to overcome the small infrarenal aorta and provide a proximal landing zone for a modified technique to preserve unilateral IIA during EVAR.

## Case series

This case series report met the exemption criteria for local ethics committee review. All patients who participated in the study provided written informed consent for the case report and imaging investigation.

### Case 1

**Fig. 1:**
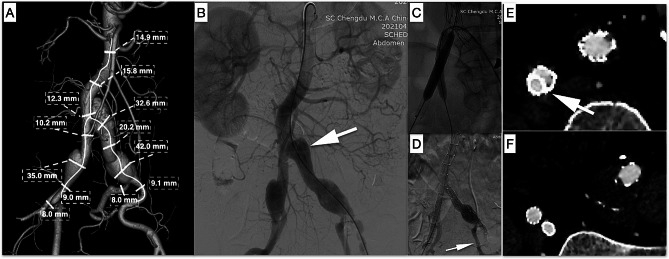
Case 1: **A** Preoperative CT imaging and aneurysm anatomical parameter measurements. The diameter of the right proximal CIA measured 10.2 mm. **B** Intraoperative angiogram. In the proximal left CIA, a dissection was observed (white arrow). **C** Two noncompliant 10 mm balloons were simultaneously inflated inside the parallel covered stents. **D** The final angiogram demonstrated successful deployment of Aegis™ and two parallel covered stents in the right iliac artery. The left IIA was embolized by coils (white arrow). **E** The cross-sectional image of the two-year follow-up CT scan demonstrated the “eye of the tiger” configuration (white arrow), with no evidence of gutter leaks. **F** On the same CT scan, the EIA and IIA stents were patent in the right iliac artery

An 83-year-old male was referred to our department for treatment of bilateral CIAAs, as well as left proximal CIA dissection (Fig. 1A, B, white arrow). Figure 1A depicts the anatomical characteristics of lesions assessed through software analysis (Endosize, Therenva SAS, Paris, France, utilized for the evaluation of all three cases). Due to his comorbid coronary artery disease and chronic obstructive pulmonary disease (COPD), he was deemed an unsuitable candidate for open surgical repair. The diameter of his right CIA was 10.2 mm, excluding him from IBD utilization (Fig. 1A). His right EIA and IIA measured 9.0 mm and 8.0 mm in diameter, respectively. We decided to proceed with coil embolization of the trunk of the left hypogastric artery, followed by EVAR utilizing the sandwich technique to preserve the right IIA.

### Case 2

**Fig. 2:**
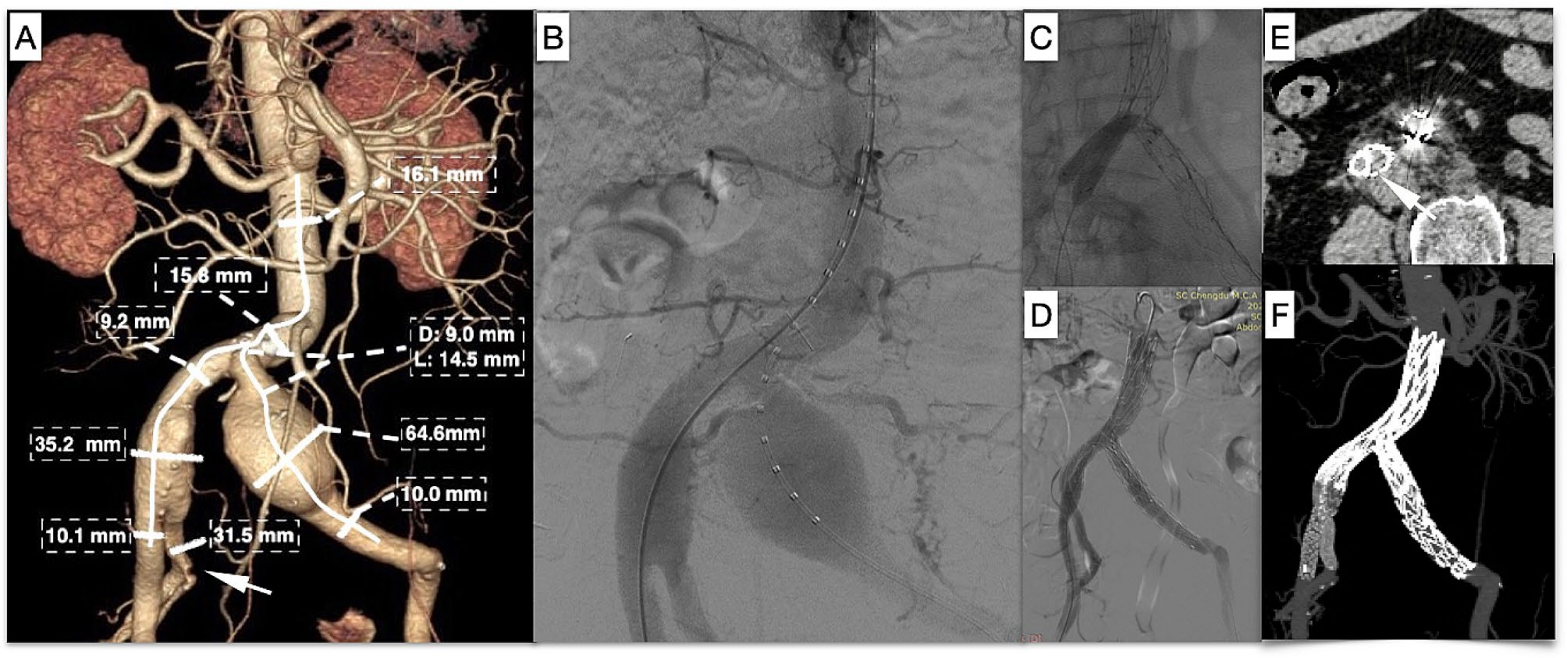
Case 2: **A** Preoperative CT imaging and aneurysm anatomical parameter measurements. The right proximal CIA measured 9.2 mm in diameter, the left proximal CIA measured 9.0 mm in diameter, and the length of the nonaneurysmal proximal CIA was 14.5 mm. **B** Intraoperative angiogram. **C** Two noncompliant 10 mm balloons were simultaneously inflated inside the parallel covered stents. **D** The final angiogram demonstrated successful deployment of Aegis™ and two parallel covered stents in the right iliac artery. **E** The cross-sectional image of the two-year follow-up CT scan demonstrated the “eye of the tiger” configuration (white arrow), with no evidence of gutter leaks. **F** CT scan three-dimensional reconstruction

A 78-year-old male with a history of hypertension and COPD was evaluated for rapidly enlarging bilateral CIAAs, the anatomical characteristics of which are depicted in Fig. 2A. The left CIAA had a diameter of 64.6 mm, while the left proximal CIA measured only 14.5 mm in length, rendering it inadequate as a proximal landing zone. His right and left CIA were approximately 9.2 mm in diameter, which was unsuitable for IBD use. As his left IIA was already occluded and his right IIA was also aneurysmal (Fig. 2A, white arrow, 31.5 mm in diameter), we planned to preserve his right gluteal artery during the EVAR procedure. The right IIA diameter was 8.8 mm at 2 cm from the aneurysm, whereas the EIA diameter was 9.6 mm at a distance of 2 cm below the bifurcation.

### Case 3

**Fig. 3:**
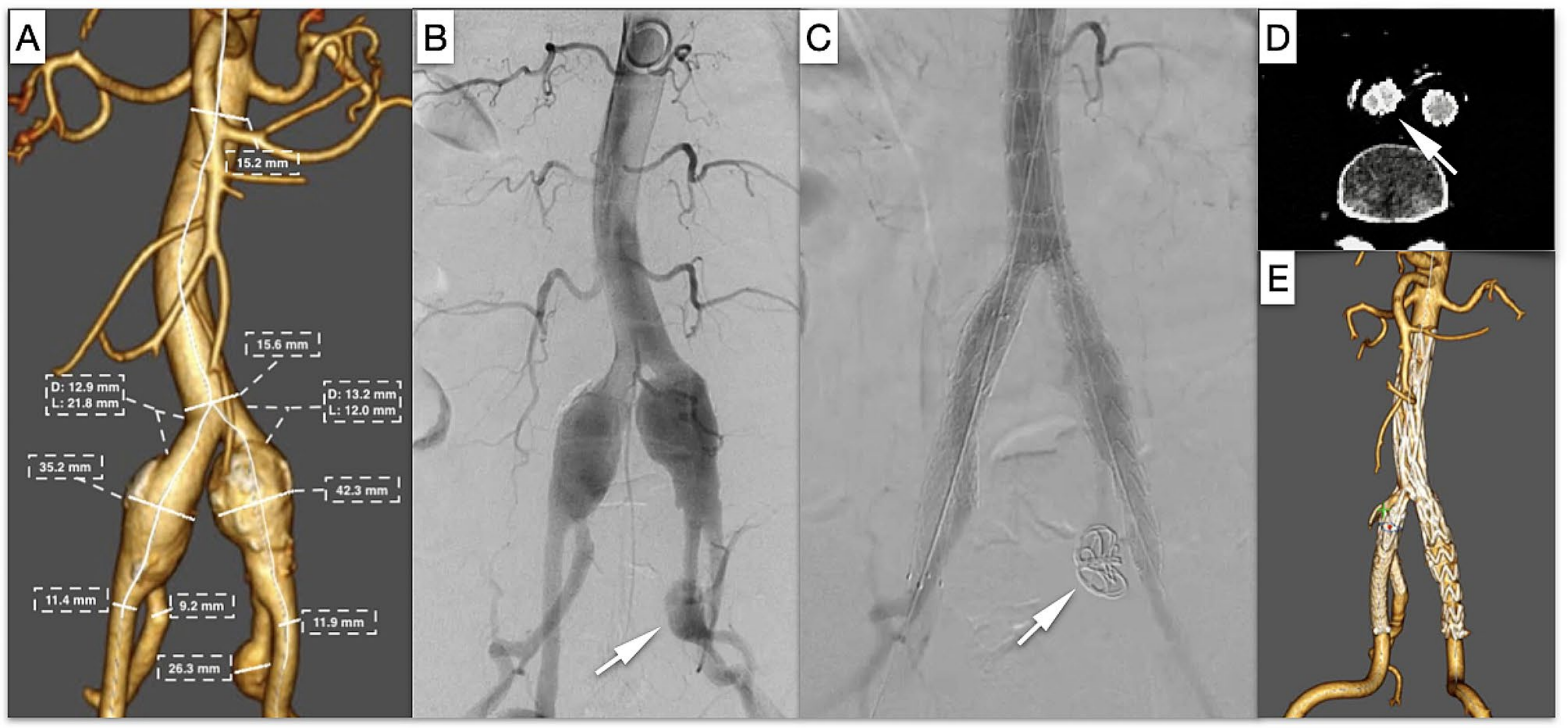
Case 3: **A** Preoperative CT imaging and measurement of aneurysm anatomical parameters. The diameter of the right proximal CIA was 12.9 mm, the diameter of the left proximal CIA was 13.2 mm, and the length of the nonaneurysmal proximal CIA was 12.0 mm. **B** Intraoperative angiogram. The left IIA was aneurysmal (white arrow), measuring 26.3 mm in diameter. **C** The final angiogram demonstrated successful deployment of Aegis™ and two parallel covered stents in the right iliac artery. The left IIA aneurysm was embolized by coils (white arrow). **E** The cross-sectional image of the two-year follow-up CT scan demonstrated the “eye of the tiger” configuration (white arrow), with no evidence of gutter leakage. **F** CT scan reconstruction in three dimensions

A 72-year-old male with bilateral CIAAs and left IIA aneurysm (Fig. 3A, B, white arrow) was referred to our department for treatment. The anatomical characteristics of the aneurysms are illustrated in Fig. 3A. The length of the right proximal CIA was 21.8 mm, and the diameter of the CIA was 12.9 mm, which was insufficient for IBD use. At 2 cm below the bifurcation, the diameter of the right EIA was 9.5 mm, while the diameter of the right IIA was 8.0 mm. His previous medical history was marked by hypertension, diabetes, and coronary artery disease. Due to his comorbid conditions, EVAR with coil embolization of the left IIA aneurysm and preservation of the right IIA was considered a treatment option.

### Surgical procedure

#### Device selection

All three cases shared a common anatomical feature, which was the absence of a suitable proximal landing zone for a covered stentgraft for at least one common iliac artery (CIA), as well as a smaller proximal CIA diameter (10.2 mm, 9.0 mm, and 12.9 mm, respectively) for IBD. Additionally, the infrarenal aorta was found to be both normal and too narrow for the implantation of a standard aortic endograft.

The Aegis™ endograft is a unibody, infrarenal bifurcated stent graft that has a main body with two attached iliac limbs [10]. The device is a walled expanded polytetrafluoroethylene (ePTFE) sheath with a self-expanding cobalt chromium alloy wire endoskeleton. The device is implanted by inserting the main body of the device through a 22 F sheath and the contralateral limb through a 7 F sheath. The contralateral limb wire is precannulated, requiring the snaring of this guidewire through the contralateral 7 F sheath. Then, the distal end of the stent is advanced to a position above the iliac bifurcation, and it can be deployed progressively.

### Stent sizing technique

In our study, we employed a modified sandwich technique wherein a balloon-expandable stent was utilized instead of a self-expandable cover stent for branch vessel reconstruction. The diameter of the IIA stent was chosen according to the size of the IIA landing zone, and the diameter of the external iliac artery (EIA) stent was chosen according to the size of the EIA landing zone and an equation that ensured that the combined cross-sectional area of the two parallel stents slightly exceeded the area of the iliac limb of Aegis™ where they were implanted [12]. The iliac limb of Aegis had a diameter of 14 mm and an area of 153.9 mm^2^. The right IIA was treated with an 8 mm X 58 mm Lifestream™ stent, which could be dilated to a diameter of 10 mm. The EIA extension was utilized with a 10 mm x 100 mm Fluency™ self-expandable covered stent-graft. The total surface area of the two stents was found to be 157.0 mm^2^, surpassing the area of 153.9 mm^2^ [2].

### Operative technique

All three procedures were conducted using local anesthesia and intravenous sedation. Right femoral access for Aegis™ introduction was obtained via surgical exposure, whereas left femoral access was obtained percutaneously. In Cases 1 and 3, the left IIA was initially embolized (Figs. 1D and 3C; white arrow) using detachable coils (Interlock™, Boston Scientific, MA, USA). Due to the normal diameter of the abdominal aorta, the smallest Aegis™ main body component (main aortic body: 22 mm in diameter and 90 mm in length; two iliac limbs: 14 mm in diameter and 40 mm in length) was chosen for all three cases. After being inserted in the conventional manner into the infrarenal aorta, the main body was released until the contralateral iliac limb was open, and the entire endograft body was pulled down to the aortic bifurcation and fully deployed.

A 7- or 8-F, 60-cm-long sheath was inserted into the right IIA using a 0.035” stiff wire (Amplatz™, Cook Medical, Bloomington, IN, USA) via the contralateral femoral access. Case 2 involved the application of a Viabahn (W.L. Gore, Flagstaff, USA) self-expandable covered stent measuring 8.0 × 50.0 mm to initially cover the aneurysm in the right IIA. In Cases 1 and 3, an 8.0 × 58.0 mm Lifestream™ stent was delivered through the sheath, at least 20 mm within the IIA and at least 30 mm proximal to the end marker of the ipsilateral limb. The Fluency™ stent was delivered via the right femoral access and positioned with a minimum 30 mm overlap with the Lifestream™ stent. In Case 2, a Lifestream™ sized 8.0 × 58.0 mm was introduced subsequent to the implantation of the Viabahn, with a 3.0 cm overlap with both devices.

The Fluency™ stent was deployed first, and then the Lifestream™ stent was inflated and deployed. Remodeling with a standard noncompliant 10 mm balloon placed inside the Fluency™ stent and the Lifestream™ stent separately to create an eyelet or “eye of the tiger” shape [13, 14] (Figs. 1E, 2E and 3D; white arrow) of the Lifestream™ stent at the overlapping zone utilizing the kiss-ballooning technique (Figs. 1C and 2C) was performed. The final angiography revealed excellent filling of the aortic endograft, both iliac limbs, and the right IIA and EIA without visualization of the bilateral CIAAs or endoleaks (Figs. 1D, 2D and 3C).

## Follow-up and results

All three patients underwent ultrasound scans at 30 days and computed tomography angiography (CTA) at 6 and 12 months and yearly thereafter for up to 5 years. The three cases were followed for twenty-four, twenty-five, and thirty-two months. In all three cases, postoperative CTA at two years revealed continued perfusion of the right IIA and bilateral EIAs. No type I or type III endoleaks were detected, and sac regression of the CIAAs was observed in all three cases (Figs. 1F, 2F and 3E).

## Discussion

The occurrence of an isolated CIAA is relatively uncommon, with a reported prevalence as low as 0.03% in autopsy studies [4]. There is a consensus that isolated CIAAs in patients at average risk should be repaired to exclude the aneurysm sac and preserve internal iliac artery perfusion [1, 7]. The literature on EVAR for the treatment of bilateral isolated CIAA is infrequent and varies according to surgeon preference, patient anatomical characteristics, and device availability [14, 15]. This case series demonstrated the efficacy and applicability of implementing an aortic bifurcated unibody endograft and modified sandwich technique as an alternative treatment option for bilateral isolated CIAAs while preserving pelvic flow.

The first report of endovascular repair of isolated CIAAs occurred in 1995[16]. Since then, this technique has undergone significant advancements and now serves as the predominant therapeutic method. In general, the available data comparing endovascular and open surgical repair indicate that endovascular repair is linked to shorter procedure durations, reduced occurrences of procedural complications, a decrease in perioperative mortality, and satisfactory long-term patency rates [17, 18]. Current guidelines strongly recommend preserving blood flow to at least one IIA during open or endovascular repair of aneurysms involving the iliac bifurcation to prevent pelvic ischemia [1].

One of the available options is hybrid procedures, which combine open surgical external-to-internal iliac artery bypass to preserve unilateral IIA flow with endovascular coverage of the IIA ostium during EVAR. Mansukhani et al. presented this technique in ten cases and established that it is well tolerated, results in a short hospital stay, and produces exceptional patency rates [19]. Several authors have demonstrated that while this technique is less invasive than open surgical aneurysm repair, it still requires general anesthesia, has longer recuperation times than EVAR alone, and cannot be performed entirely percutaneously [12, 20].

In the context of the EVAR procedure, there are a variety of techniques with varying degrees of efficacy for preserving flow to the IIA. “Bell-bottom” or flared iliac stent grafts are one of the most frequently employed techniques for preserving pelvic flow in patients with small CIA aneurysms [21]. With 28-mm diameter flared iliac stent graft implantation, the maximum acceptable iliac diameter increased to 25 mm, allowing for a reliable seal in the aneurysmal segment of the iliac artery. However, the application of this technique has been limited due to the size of the stent graft and anatomical constraints, as well as the potential risk of type Ib endoleak, which could result from expansion of the aneurysm over time and increase the need for a secondary intervention [22].

Commercialized iliac branch devices (IBDs) for the exclusion of aorto-iliac or isolated CIA aneurysms have been developed in recent years to preserve pelvic circulation. In the first prospective multicenter study of 52 patients with CIAAs treated with IBDs combined with different aortic components, Haulon et al. [23] reported CIA shrinkage in 81% of patients at the 1-year follow-up and iliac branch occlusion in 11% of cases within the first month. Several research investigations have demonstrated outstanding early and midterm safety and efficacy of IBDs in treating aorto-iliac aneurysms or isolated CIAAs, as well as low rates of complications and mortality [7, 24]. These results support the use of IBDs as the primary treatment option for patients with intra-abdominal aneurysms and a suitable vascular anatomy [15, 25]. IBDs can be used either in combination with an aortic stent-graft or as a standalone treatment, as specified in the instructions for use (IFU). The main challenges of this procedure are its high cost, long duration, and increased complexity. Furthermore, there are specific anatomical prerequisites for IBD treatment. Several analysts have suggested that strictly following the manufacturer’s IFU would limit the eligibility of patients to only approximately one-third of the total population [9, 14].

Several studies [26–28] have identified a smaller proximal common iliac artery (CIA) diameter as one of the most prevalent reasons for not meeting IFU recommendations in East Asian populations. However, these studies [27, 28] have also determined that a diameter > 13 mm is acceptable for the use of Gore iliac branch endoprostheses, in contrast to the IFU recommendation of a diameter > 17 mm. In one case in their study [28], iliac limb stenosis was treated with stenting 11 days after surgery due to a proximal CIA diameter of 11 millimeters. In our case series, the proximal CIA diameter was 10.2 mm in Case 1, 9.0 mm in Case 2, and 12.9 mm in Case 3. These findings suggest a potential risk of iliac limb stenosis associated with the use of IBDs.

When commercial IBDs are deemed inappropriate for CIAA management, custom IBDs are available as an alternative. Although a restricted number of studies [29, 30] have provided evidence regarding the safety and effectiveness of this method, there is a scarcity of substantiating evidence to support its application.

The “sandwich” technique was first described by Lobato [31], who used parallel covered self-expandable stent-graft implants to preserve blood flow into one or both IIAs. Using this technique, the iliac limb of the aortic stent graft terminated just above the bifurcation in the CIA; two overlapping stents were implanted into the lower portion of the iliac limb, extending into the EIA and IIA to maintain blood flow. Recent publications have confirmed this technique’s excellent patency and low endoleak rates in short- and medium-term follow-up, although there was less clinical evidence than for IBD [32, 33]. A similar technique with the use of an Aorfix stent-graft was described by Lim et al. in a series of 21 patients (25 iliac aneurysms) treated using balloon and self-expandable endovascular prostheses implanted in hypogastric and external iliac arteries [34]. These outcomes are similarly remarkable. This technique has several limitations, including the potential for compression of one of the parallel grafts, type III endoleaks, and a paucity of large-sample controlled long-term data on limb occlusion and endoleaks.

To address the problems of stent compression or type III endoleaks, some equations based on the circumference or cross-sectional area calculation have been generated to select the right size of parallel stents [12, 35]. In addition, there has been a progression away from the traditional “sandwich technique”, where stents are typically self-expandable. Minion [13] proposed the conversion of round grafts into eye-shaped grafts through the expansion of parallel grafts, known as the “eye of the tiger” technique. This technique reduces the gutter between parallel grafts and enhances sealing effectiveness. Several authors have proposed the utilization of balloon-expandable stents instead of self-expandable stents for the application of this modified technique [14, 36]. This approach offers the advantage of facilitating sizing and enables the balloon-expandable nature of the IIA stent to fully fill the gutters in a standard sandwich technique. During the time frame of our case series, Lifestream™ was the only balloon-expandable covered stent available in China. The utilization of Lifestream™ for the sandwich technique in preserving the IIA has been infrequently documented in the literature. However, Bertoglio et al. conducted a study where they employed Lifestream™ in fenestrated and branched thoracoabdominal endovascular repairs. They observed favorable patency rates but unexpectedly encountered a high incidence of type III endoleaks in the one-year follow-up period. It was hypothesized that a lack of sufficient radial force to obtain/maintain an effective seal after flaring could be the cause [37]. However, we did not observe this phenomenon in our sandwich technique case series since we overdilated the 8 mm Lifestream™ to 10 mm. The results of the two-year follow-up revealed favorable patency rates for both dual parallel stents, with no instances of endoleaks observed. The subsequent CT scan revealed that the final configuration, when viewed in cross section, bore a resemblance to the “eye of the tiger” pattern, characterized by one edge exhibiting a D shape and the other edge displaying a flared contour that resulted in the elimination of the gutters (Figs. 1E, 2E and 3D, white arrow).

For isolated CIAAs, if the proximal landing zone is insufficient for the IBD or covered stent, an aortic stent-graft is typically utilized to provide a landing zone for the IBD or covered stent. In the majority of instances, the abdominal aorta was afflicted with aneurysmal changes, allowing for the use of standard bifurcated aortic endografts. When the infrarenal abdominal aorta was normal, this was considered a possible contraindication for the utilization of bifurcated aortic endografts. Current guidelines recommend an aortic bifurcation diameter of > 20 mm for the use of a bifurcated graft to prevent limb occlusion caused by limb competition within the narrow distal aorta [1]. Under such circumstances, bifurcated unibody endografts may be utilized. Another advantage of the bifurcated unibody endograft is the ability to place the IIA stent graft from the contralateral groin without the need for brachial access. DeRubertis et al. [12] reported the application of the Endologix AFX2™ bifurcated unibody device to perform the sandwich technique to preserve blood flow to the IIA. They discovered that this reduced the technical complexity of these procedures and decreased the morbidity associated with brachial access. Akagi et al. [38] also recently presented two cases in which they effectively preserved hypogastric blood flow and overcame anatomical limitations by combining AFX2™ and IBD. We utilized an Aegis™ bifurcated unibody endograft to circumvent the small infrarenal aorta and completed the entire operation without brachial access. We discovered that the 7- or 8-F sheath could readily traverse the main body of the endograft and be delivered into the IIA via contralateral femoral access, thereby reducing the time needed to insert the stent.

Our study was limited by a two-year follow-up period; therefore, an extended follow-up period is needed to verify the efficacy and safety of this combination of bifurcated unibody endografts and the modified sandwich technique. Recent studies have recommended maintaining the bilateral IIA, which is another drawback of this study [8]. For Cases 1 and 3, although they did not develop pelvic ischemia symptoms, the same technique could be used to preserve the other IIA, although economic considerations must be taken into account.

## Conclusion

This study demonstrated that using an aortic bifurcated unibody endograft and modified sandwich technique was a viable and secure method for preserving pelvic blood flow in patients with bilateral isolated CIAAs. This approach was particularly effective for patients who did not have a suitable landing zone, had a normal diameter of the proximal CIA, and had a normal small infrarenal aorta. These patients were not eligible for iliac branch devices (IBDs) or standard aortic endograft implantation. The initial outcomes of this technique showed promise. Future research should prioritize long-term outcomes and carefully select appropriate patients for the application of this technique.

## Data Availability

All patient information was stored in the electronic record system and the PACS system of the two institutions (Hospital of Chengdu University of Traditional Chinese Medicine and Panzhihua Municipal Central Hospital); if necessary, we can provide patient-specific details without disclosing their personal information.
